# Structural brain morphometry differences and similarities between young patients with Crohn’s disease in remission and healthy young and old controls

**DOI:** 10.3389/fnins.2024.1210939

**Published:** 2024-01-31

**Authors:** Benjamin Yeske, Jiancheng Hou, Daniel Y. Chu, Nagesh Adluru, Veena A. Nair, Poonam Beniwal-Patel, Sumona Saha, Vivek Prabhakaran

**Affiliations:** ^1^School of Medicine and Public Health, University of Wisconsin-Madison, Madison, WI, United States; ^2^Center for Cross-Straits Cultural Development, Fujian Normal University, Fuzhou City, Fujian, China; ^3^Department of Radiology, School of Medicine and Public Health, University of Wisconsin-Madison, Madison, WI, United States; ^4^Neuroscience Training Program, University of Wisconsin-Madison, Madison, WI, United States; ^5^The Waisman Center, University of Wisconsin-Madison, Madison, WI, United States; ^6^Gastroenterology and Hepatology, Department of Medicine, Medical College of Wisconsin, Milwaukee, WI, United States; ^7^Gastroenterology and Hepatology, Department of Medicine, University of Wisconsin- Madison, Madison, WI, United States; ^8^Department of Psychology and Psychiatry, University of Wisconsin-Madison, Madison, WI, United States

**Keywords:** Crohn’s disease, IBD, structural imaging, cognitive function, gut-brain axis, aging

## Abstract

**Introduction:**

Crohn’s disease (CD), one of the main phenotypes of inflammatory bowel disease (IBD), can affect any part of the gastrointestinal tract. It can impact the function of gastrointestinal secretions, as well as increasing the intestinal permeability leading to an aberrant immunological response and subsequent intestinal inflammation. Studies have reported anatomical and functional brain changes in Crohn’s Disease patients (CDs), possibly due to increased inflammatory markers and microglial cells that play key roles in communicating between the brain, gut, and systemic immune system. To date, no studies have demonstrated similarities between morphological brain changes seen in IBD and brain morphometry observed in older healthy controls..

**Methods:**

For the present study, twelve young CDs in remission (M = 26.08 years, SD = 4.9 years, 7 male) were recruited from an IBD Clinic. Data from 12 young age-matched healthy controls (HCs) (24.5 years, SD = 3.6 years, 8 male) and 12 older HCs (59 years, SD = 8 years, 8 male), previously collected for a different study under a similar MR protocol, were analyzed as controls. T1 weighted images and structural image processing techniques were used to extract surface-based brain measures, to test our hypothesis that young CDs have different brain surface morphometry than their age-matched young HCs and furthermore, appear more similar to older HCs. The phonemic verbal fluency (VF) task (the Controlled Oral Word Association Test, COWAT) (Benton, 1976) was administered to test verbal cognitive ability and executive control.

**Results/Discussion:**

On the whole, CDs had more brain regions with differences in brain morphometry measures when compared to the young HCs as compared to the old HCs, suggesting that CD has an effect on the brain that makes it appear more similar to old HCs. Additionally, our study demonstrates this atypical brain morphometry is associated with function on a cognitive task. These results suggest that even younger CDs may be showing some evidence of structural brain changes that demonstrate increased resemblance to older HC brains rather than their similarly aged healthy counterparts.

## Introduction

Crohn’s disease (CD), one of the main phenotypes of inflammatory bowel disease (IBD), can affect any part of the gastrointestinal tract (Fiorindi et al., 2022; Godala et al., 2022). It can impact the function of gastrointestinal secretions, as well as increasing the intestinal permeability leading to an aberrant immunological response and subsequent intestinal inflammation (Chichlowski and Hale, 2008). Even patients in remission experience post-inflammatory changes leading to intestinal hypersensitivity (Jacobson et al., 1995). There is evidence suggesting that the inflammatory response of IBD may affect a patient’s mental state by altering motor and sensory systems causing difficulties with cognition (Nair et al., 2016; van Langenberg et al., 2017; Sharma et al., 2021) and psychological stress precipitating mood disorders (Zhang et al., 2016; Banovic et al., 2020). The effect of IBD may also alter the brain and lead to anatomical and functional changes. Several studies have reported anatomical and functional brain changes in Crohn’s Disease patients (CDs), possibly due to increased inflammatory markers and microglial cells that play key roles in communicating between the brain, gut, and systemic immune system (Sajadinejad et al., 2012; Hou et al., 2019). It has been proposed that these systemic alterations lead to a series of changes to neuronal connections and processes resulting in anatomical or functional brain changes that impact cognitive or emotion regulation skills (Zeng et al., 2012; Nair et al., 2016; Bao et al., 2017b). These brain changes may also explain why CDs tend to have a reduced ability to regulate cognitive and emotional states than their non-CD counterparts (Thomason and Thompson, 2011; Bushnell et al., 2013; Nair et al., 2016). Additionally, anatomical and functional changes in the brain may be influenced by the comorbidities associated with CD such as chronic pain, psychological stress, anxiety, and depression (Sajadinejad et al., 2012).

There is mounting evidence suggesting that the differences observed in brain function and structure of CDs may be correlated with cognitive differences. For instance, a couple of diffusion tensor imaging (DTI) studies have identified white matter (WM) microstructural differences in CDs compared to heatlhy controls (HCs). Zikou et al. reported IBD patients (CD or ulcerative colitis) who showed decreased axial diffusivity in the right corticospinal tract (involved in motor function) and right superior longitudinal fasciculus (involved in language function) when compared to HCs (Zikou et al., 2014). Our previous DTI study identified significant alterations in WM microstructure of CDs compared to HCs in brain regions implicated in language function despite the absence of differences in a verbal fluency measure designed to assess verbal cognitive ability and executive control (Hou et al., 2020).

A meta-analysis of CDs brain imaging literature reported reduced GM volume in the medial frontal gyrus compared to that of HCs (Yeung, 2021). Bao, et al. identified cortical thickness of the left insula and orbitofrontal cortex and gray matter (GM) volumes of the right anterior cingulate cortex (ACC), dorsomedial prefrontal cortex and left insula were negatively correlated with disease duration (Bao et al., 2015). A subsequent study by Bao, et al. identified differences in GM volumes between CDs in remission with and without abdominal pain, finding lower GM volumes in the insula and ACC in CDs with pain compared to those without (Bao et al., 2017a). Other regions of cortical thickness increases, and sub-cortical volume decreases, have also been reported and correlated to pain score or disease duration (Nair et al., 2016). Zikou et al. also found brain regions of atrophy in CDs such as the bilateral fusiform and inferior temporal gyrus which are related to emotion processing (Zikou et al., 2014). A study by Thapaliya, et al., demonstrated a significant reduction in gray matter volume (GMV), white matter volume and cortical thickness in the left prefrontal gyrus and increased GMV in frontal brain regions in CDs versus HCs (Thapaliya et al., 2022). Additionally, another study found CDs with extraintestinal manifestations of the disease, but not those without such manifestations, were especially prone to cortical brain changes, suggesting that brain changes are more strongly influenced by the systemic inflammation of the disease (Thomann et al., 2016).

Our previous task-based functional magnetic resonance imaging study looking at verbal fluency of CDs in remission found that activity intensity in regions of the right hemisphere was positively correlated with disease duration. Furthermore, the study identified similar task activation patterns between young adult CDs and healthy older HCs. This suggests that young adult CD brain changes may resemble brains older healthy adults (Nair et al., 2019), perhaps due to the increase of proinflammatory cytokine exposure in both aging adults and CDs. Additionally, IBD has been associated with age-related diseases such as, Parkinson’s (Lin et al., 2016; Brudek, 2019; Zeng et al., 2022) and Alzheimer’s disease (Hillary et al., 2020; Wang et al., 2022). To date, no studies have demonstrated similarities between morphological brain changes seen in IBD and brain morphometry observed in older healthy controls.

Among many techniques, the brain cortical thickness measures using magnetic resonance imaging (MRI) have proven sensitive to examine the changes in brain structure and development with some studies having used the volumetric measurement (e.g., voxel-based morphometry) to examine CD in remission (Nair et al., 2016; Thomann et al., 2021). However, volumetric measurement has some limitations. For example, it is inadequate for investigating brain surface folding due to its lack of statistical power (Lemaitre et al., 2012; Jin et al., 2018). Other cortical surface morphometries such as the cortical thickness, fractal dimensionality (FD), gyrification, and sulcal depth also influence the volumetric results (Trost et al., 2013; Hirakawa et al., 2016).

Cortical thickness measures the distance between the points on the pial and white matter boundaries of the neocortex, in addition to measuring the gray matter morphological difference (Hirakawa et al., 2016; Seiger et al., 2018). However, cortical thickness is limited to the cortex and therefore it cannot examine non-cortical regions (Bermudez et al., 2009). Another measure, fractal dimensionality, reflects how the brain structure fits to space constraints (Yotter et al., 2011b) and is used to investigate cortical complexity of cerebral folding reported as a single numerical value (Di Ieva et al., 2014, 2015; Madan and Kensinger, 2017). Studies have demonstrated that FD is sensitive to internal shape complexity of the brain that gray matter volume and cortical thickness measures are not (Zhang et al., 2008; Madan and Kensinger, 2017; Chen et al., 2020). Gyrification examines the level of local cortical folding that relates to the integrity between subcortical and cortex circuits (Li et al., 2021). Sulcal depth, based on the Euclidean distance between the pial and outer surface (Yun et al., 2013; Li et al., 2021), is generated from the changes of gray and white matter in the cerebral cortex as well as subcortical structures, making it sensitive to the complicated folding of the cerebral surface (Im et al., 2008; Kim et al., 2008; Kochunov et al., 2008; Jin et al., 2018).

In the current study, we aim to build upon our previous study by using structural imaging techniques that include the cortical thickness, fractal dimensionality, gyrification, and sulcal depth (see Methods for description of each metric), to test our hypothesis that the young CDs have different brain surface morphometry than their age-matched young HCs and furthermore, appear more similar to the older HCs. Additionally, we hypothesize these structural changes will be reflected in functional outcome differences in cognitive function.

## Methods

### Participants

Twelve young CDs in remission (*M* = 26.08 years, SD = 4.9 years, 7 male) were recruited from the IBD Clinic. Data from 12 young age-matched HCs (24.5 years, SD = 3.6 years, 8 male) and 12 older HCs (59 years, SD = 8 years, 8 male), previously collected for a different study with similar MR scan protocol, were analyzed as controls. Participant characteristics are shown in Table 1. HCs had no history of substance abuse, affective, psychiatric, or neurological disorders, and were mostly right-handed (Oldfield, 1971). The participants were screened for cognitive deficits using the Mini-Mental State Examination (Folstein et al., 1975) and provided written informed consent. The protocol was reviewed and approved (#H2014–0131) by the local health sciences IRB. All methods were carried out in accordance with relevant guidelines and regulations. All experimental protocols were approved by the Institutional Review Board (IRB) of the School of Medicine and Public Health, University of Wisconsin-Madison.

**Table 1:** Characteristics and cognitive measures among young CDs, young HCs and old HCs.

Characteristics	Young CDs	Young CDs	Old CDs	*F*_(2, 33)_	*p*
Number	12	12	12		
Age (years)	26.083 (4.926)	24.500 (3.606)	59.250 (8.081)	135.145	0.000
Education	15.750 (2.563)	16.417 (2.021)	16.583 (2.275)	0.442	0.646
Gender (male/female)	7/5	8/4	8/4	0.111	0.895
Handedness (L/R/A)	1/8/3	1/11/0	0/12/0	2.029	0.148
Mean VF raw score	45.417 (13.222)	45.250 (11.702)	42.417 (9.424)	0.255	0.776
IBD medications	Antibiotics 0, 5- aminosalicyclate, 7 immunomodulator 6, antitumor necrosis factorα 9, anti-integrin1, corticosteroids 0				

### Behavioral data acquisition

We administered the phonemic verbal fluency (VF) task (the Controlled Oral Word Association Test, COWAT; Benton, 1976) to test verbal cognitive ability and executive control. All CDs and HCs were tested for the VF task outside the scanner. COWAT has been extensively used in both clinical and non-clinical populations on account of its face validity (Sauzéon et al., 2011), assessment of both verbal cognitive ability and executive control (Fisk and Sharp, 2004), and high correlation with measures of attention, verbal memory, and word knowledge (Ruff et al., 1997). Participants were required to produce words beginning with the letters “F,” “A,” “S,” in three 1-min trials, respectively. A normalized VF *z*-score, corrected for age and education, based on the total correct responses over the 3 trials was used to quantify VF performance for each participant.

### MRI data acquisition

The MRI data were acquired on a GE750 3 T MRI scanner. A whole brain high-resolution 3D T1-weighted BRAVO, IR-prepared FSPGR (Fast Spoiled Gradient Recalled Echo), MRI sequence with 156 axial slices was performed for each participant using the following parameters: TR = 8.132 ms, TE = 3.18 ms, TI = 450 ms, feld of view = 256 × 256 mm^2^, flip angle = 12, matrix = 256 × 256, in-plane resolution =1 × 1 mm^2^, slice thickness = 1.0 mm.

### Cortical surface preprocessing

The Computational Anatomy Toolbox (CAT12)^1^, which is a plug-in software based on Statistical Parametric Mapping (SPM12)^2^ and integrated into MATLAB (MathWorks), was used for the T1-weighted MRI data preprocessing. The CAT12 is not only a more precise and accurate analysis of gray matter volume than the previous voxel-based morphometry plug-in toolbox in SPM (Farokhian et al., 2017; Yuksel et al., 2018), but also is fully automated for surface-based analysis (Zhuang et al., 2017). The data preprocessing with CAT12 consisted of bias-field correction, skull-stripping, and alignment to the Montreal Neurological Institute (MNI) structural template to classify gray matter (GM), white matter (WM) and cerebrospinal fluid (CSF), as well as spatial normalization with the Diffeomorphic Anatomical Registration Through Exponentiated Lie Algebra (DARTEL) registration (1.5 mm) (Kurth et al., 2015; Zhuang et al., 2017; Yuksel et al., 2018). Subsequently, we employed a spherical harmonic method (Yotter et al., 2011a) to reparametrize the cortical surface mesh based on an algorithm that reduces area distortions (Yotter et al., 2011c) to repair any topological defects (Yotter et al., 2011a,c; Chen et al., 2020). Cortical thickness was analyzed based on the workflow specified in the study by Dahnke et al. (2013). This algorithm uses tissue segmentation to evaluate the WM distance and also projects the local maxima to other GM voxel. Values at the outer GM boundary in the WM distance map is projected back to the inner GM boundary to generate the GM thickness (Li et al., 2021). Following this, a central surface was created at the 50% level of the percentage position between the WM distance and GM thickness (Li et al., 2021). For the resultant central surface, a topology correction based on spherical harmonics was used to account for topological defects (Yotter et al., 2011a; Li et al., 2021). Moreover, the central surface was reparameterized into a common coordinate system through spherical mapping, and the spatial normalization was used with the DARTEL registration (Li et al., 2021). Spatially smoothing with 15 mm full width at half maximum (FWHM) Gaussian kernel was used for this analysis.

The fractal dimensionality estimates cortical fold complexity based on spherical harmonic reconstructions (Yotter et al., 2011a; Li et al., 2021) and is calculated as the slope of a logarithmic plot of surface area versus the maximum l-value, where the maximum l-value is a measure of the bandwidth of frequencies used to reconstruct the surface shape (Yotter et al., 2011b; Li et al., 2021). Smoothing with 15 mm FWHM Gaussian kernel was used for the fractal dimensionality analysis.

Based on the spherical harmonic reconstructions, the gyrification, as an indicator of cortical folding, was calculated as absolute mean curvature (Luders et al., 2006; Li et al., 2021). Mean curvature is an extrinsic surface measure, and provides information about the change in normal direction along the surface (Li et al., 2021). Smoothing with 15 mm FWHM Gaussian kernel was used for this analysis.

The sulcal depth measures the depth of sulci and is calculated as the Euclidean distance between the central surface and its convex hull based on the spherical harmonic reconstructions, then transformed with the sqrt function (Li et al., 2021). Smoothing with 15 mm FWHM Gaussian kernel was used for this analysis.

### Statistical analysis

The demographic differences between the CDs and young or old HCs were analyzed by independent samples *t*-tests. Group comparisons of cortical thickness, fractal dimensionality, gyrification, and sulcal depth were performed using the CAT12 and analyzed via a non-parametric permutation technique. The Threshold-Free Cluster Enhancement (TFCE) was used in permutation testing with 5,000 permutations (Smith and Nichols, 2009). TFCE *p* < 0.05 images obtained were family-wise error corrected for multiple comparisons across space. The brain regions with cluster size at least 100 vertices (cluster size × percentage covered in the specific region produced by CAT12) were reported. The Desikan–Killiany atlas (DK40) (Desikan et al., 2006) was used to label the cortical regions and the results were visualized using the CAT12. Moreover, when group differences with detailed regions were observed in CAT12, we conducted the Pearson correlation between each surface index and VF score in each group in IBM SPSS version 23, with its threshold of family-wise error corrected *p* < 0.05.

## Results

### Behavior

A One-Way ANOVA showed that there were no significant differences between young CDs, young HCs, and old HCs on education, VF score, gender and handedness. Posthoc analysis revealed no age difference between young CDs and young HCs (*p* = 0.512), but there was a statistical difference between the ages of young CDs and old HCs (*p* = 0.000), and also between the young HCs and old HCs (*p* = 0.000) (see Table 1 for details).

### Group differences in cortical surface measures

#### Cortical thickness

**Figure 1:**
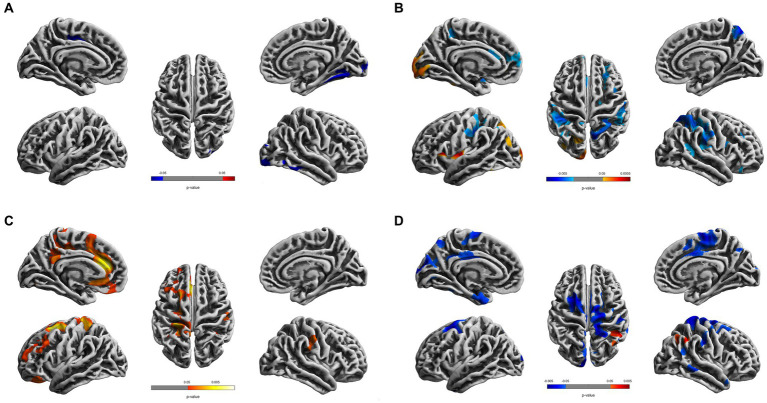
Cortical surface differences between young CDs and young HCs. Non-parametric permutation testing with 5,000 permutations and threshold-free cluster enhancement (TFCE) with family-wise error corrected threshold of *p* < 0.05 was used. Red: younger CDs increased sulcal depth compared to younger HCs. Blue: younger CDs decreased sulcal depth compared to younger HCs. **(A)** Cortical thickness. **(B)** Fractal dimensionality. **(C)** Gyrification **(D)** Sulcal depth.

**Figure 2:**
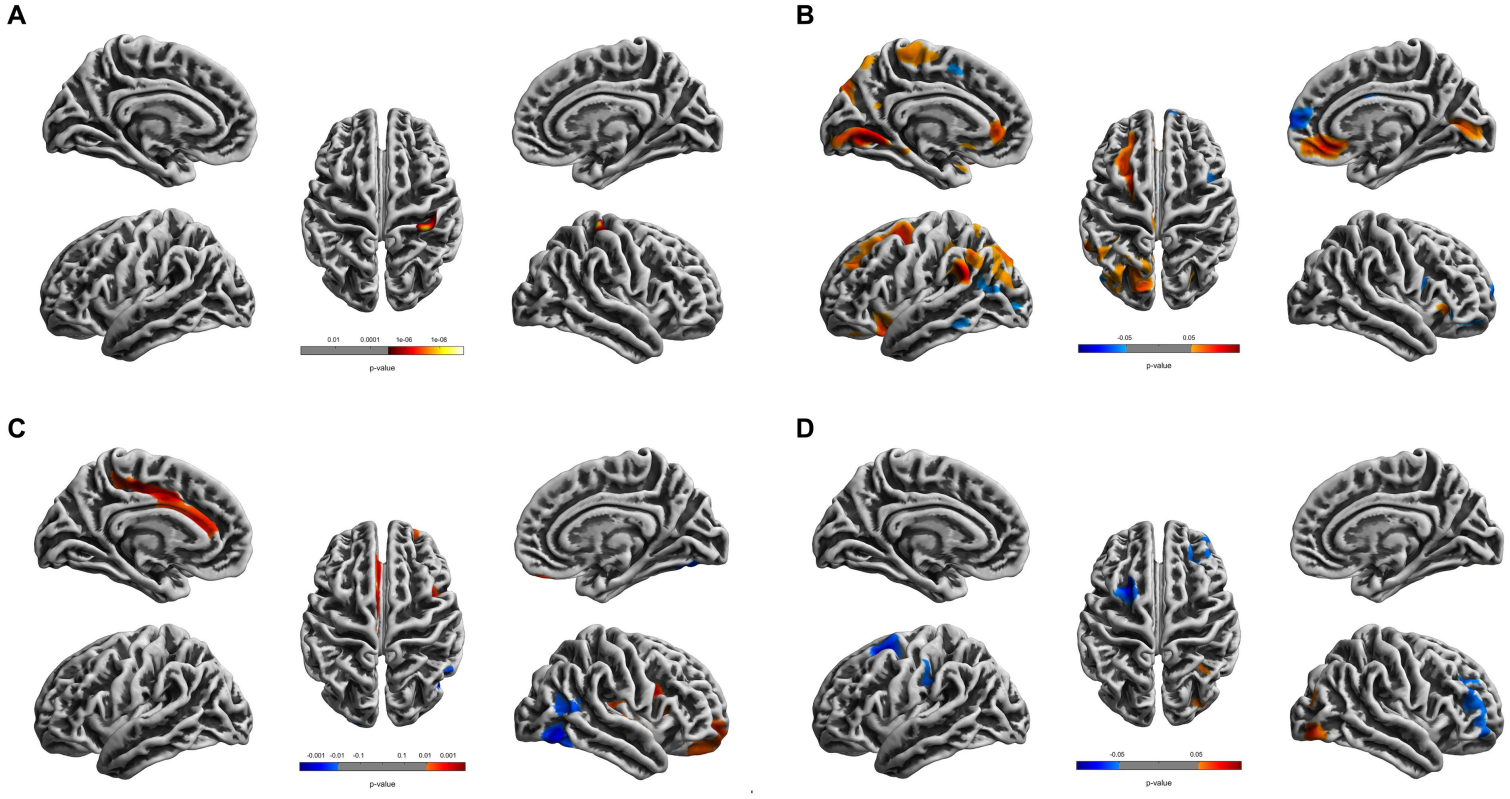
Cortical surface differences between younger CDs and older HCs. Non-parametric permutation testing with 5,000 permutations and threshold-free cluster enhancement (TFCE) with a family-wise error correction threshold of *p* < 0.05 was used. Red: younger CDs increased sulcal depth compared to older HCs. Blue: younger CDs decreased sulcal depth compared to older HCs. **(A)** Cortical thickness. **(B)** Fractal dimensionality. **(C)** Gyrification. **(D)** Sulcal depth.

Compared to the young HCs, the young CDs demonstrated significantly decreased cortical thickness in the right fusiform, inferior occipital and lingual gyri (see Figure 1A and Table 2). However, the young CDs exhibited significantly increased cortical thickness in the right postcentral gyrus compared to the old HCs (see Figure 2A and Table 3).

**Table 2:** Differences of cortical surface measures between young CDs and young healthy controls.

Group differences	Regions	Coordinates			Peak *t*-value	*p*	Cluster size
		*x*	*y*	*z*			
	*Thickness (right hemisphere)*						
CDs < Controls	Fusiform	41	−12	−28	2.47	0.022	150
	Lateral occipital	44	−69	29	2.40	0.024	212
	Lingual gyrus	26	−59	5	2.69	0.031	100
	*Fractal dimensionality (left hemisphere)*						
CDs > Controls	Lateral occipital gyrus	−9	−102	9	3.77	0.004	201
	Lingual gyrus	−54	−35	−9	2.42	0.004	147
	Superior parietal lobule	−19	−67	42	2.64	0.018	246
	Inferior parietal lobule	−30	−69	32	2.58	0.018	145
	Insula	−35	5	5	4.37	0.000	222
CDs < Controls	Supramarginal gyrus	−56	−47	26	2.45	0.003	434
	Postcentral gyrus	−33	−24	14	2.23	0.003	319
	Superior temporal gyrus	−47	−39	−25	2.75	0.008	173
	Superior frontal gyrus	−23	2	49	2.13	0.038	118
	*Fractal dimensionality (right hemisphere)*						
CDs < Controls	Supramarginal gyrus	56	−47	35	2.61	0.001	750
	Superior parietal lobule	32	−50	62	2.53	0.001	673
	Inferior parietal lobule	54	−59	6	2.89	0.035	119
	Postcentral gyrus	56	−4	21	2.42	0.001	517
	Precuneus	53	−60	−4	2.83	0.001	155
	Insula	39	−5	4	2.53	0.001	155
	Parstriangularis gyrus	38	13	−29	3.29	0.001	103
	Rostral middle frontal gyrus	35	37	−8	2.55	0.011	238
	*Gyrification (left hemisphere)*						
CDs > Controls	Superior frontal gyrus	−21	9	59	5.74	0.002	432
	Rostral middle frontal gyrus	−27	34	25	2.46	0.030	168
	Medial orbitofrontal gyrus	−14	35	−24	2.71	0.002	228
	Lateral orbitofrontal gyrus	−7	7	47	2.41	0.002	132
	Caudal anterior cingulate gyrus	−8	37	16	4.25	0.002	191
	Rostral anterior cingulate gyrus	−3	−12	30	2.23	0.002	191
	Isthmus cingulate gyrus	−6	−48	31	2.91	0.020	143
	Superior parietal lobule	−42	−67	45	2.82	0.006	127
	Paracentral lobule	−7	−22	51	2.91	0.010	167
	Postcentral gyrus	−28	−33	70	3.54	0.006	334
	Precentral gyrus	−6	−3	70	2.86	0.006	140
	Precuneus	−16	−42	57	2.52	0.010	131
	*Gyrification (right hemisphere)*						
CDs > Controls	Postcentral gyrus	57	−16	42	3.07	0.019	156
	Supramarginal gyrus	58	−25	25	2.55	0.019	123
	*Sulcal depth (left hemisphere)*						
CDs < Controls	Superior frontal gyrus	−20	42	34	2.23	0.007	426
	Caudal middle frontal gyrus	−11	46	10	2.21	0.007	187
	Posterior cingulate gyrus	−4	−42	70	2.33	0.024	221
	Cuneus	−17	−79	43	2.76	0.010	172
	Entorhinal gyrus	−51	−20	−36	2.87	0.026	169
	*Sulcal depth (right hemisphere)*						
CDs > Controls	Inferior parietal lobule	22	−50	43	2.15	0.009	206
CDs < Controls	Precentral gyrus	5	−58	34	2.23	0.008	375
	Paracentral lobule	9	−33	49	2.14	0.008	324
	Superior parietal lobule	21	−62	62	2.40	0.008	273
	Inferior parietal lobule	51	−49	40	3.46	0.036	117
	Superior frontal gyrus	44	26	25	2.23	0.008	256
	Precentral gyrus	55	4	41	2.13	0.036	110
	Postcentral gyrus	44	−25	47	2.35	0.008	222
	Posterior cingulate gyrus	14	−37	41	2.40	0.008	171
	Supramarginal gyrus	55	−37	30	2.07	0.014	203
	Middle temporal gyrus	64	−10	−20	2.94	0.025	122

N-parametric permutation testing with 5,000 permutations and threshold-free cluster enhancement (TFCE) with family-wise error corrected threshold of *p* < 0.05 was used.

**Table 3:** Differences of cortical surface measures between young CDs and old healthy controls.

Group differences	Regions	Coordinates			Peak *t* value	*p*	Cluster size
		*x*	*y*	*z*			
	*Thickness (right hemisphere)*						
CDs > Controls	Postcentral gyrus	36	−35	66	2.65	0.000	221
	*Fractal dimensionality (left hemisphere)*						
CDs > Controls	Superior frontal gyrus	−22	3	50	3.43	0.004	391
	Rostral middle frontal gyrus	−27	15	42	2.72	0.004	211
	Caudal middle frontal gyrus	−22	40	34	2.30	0.004	180
	Lateral orbitofrontal gyrus	−13	36	−23	2.31	0.008	108
	Superior parietal lobule	−31	−73	44	2.41	0.010	370
	Superior parietal lobule	−35	−45	39	2.27	0.027	116
	Paracentral lobule	−7	−26	59	2.49	0.020	203
	Lingual gyrus	−12	−66	−3	3.65	0.002	427
	Supramarginal gyrus	−56	−47	26	4.31	0.001	268
	Supramarginal gyrus	−59	−21	27	2.81	0.011	139
	Rostral anterior cingulate gyrus	−2	−20	28	3.37	0.006	126
CDs < Controls	Inferior parietal lobule	−4	−37	25	2.89	0.022	139
	*Fractal dimensionality (right hemisphere)*						
CDs > Controls	Pericalcarine	18	−71	10	2.57	0.011	259
	Medial orbitofrontal gyrus	8	32	−12	3.62	0.004	235
CDs < Controls	Superior frontal gyrus	20	33	51	2.49	0.008	139
	Parstriangularis gyrus	64	−6	11	2.32	0.023	101
	*Gyrification (left hemisphere)*						
CDs > Controls	Posterior cingulate gyrus	−12	−21	39	3.84	0.000	307
	Caudal anterior cingulate gyrus	−4	25	18	4.34	0.000	233
	Paracentral lobule	−21	−47	62	3.89	0.000	223
	*Gyrification (right hemisphere)*						
CDs > Controls	Superior temporal gyrus	25	−7	−30	3.19	0.000	197
	Insula	41	−5	3	2.59	0.000	158
	Rostral middle frontal gyrus	39	8	24	4.43	0.002	118
	Lateral orbitofrontal gyrus	20	56	−8	3.77	0.002	108
	Fusiform	33	−42	−16	4.03	0.001	172
	Lateral occipital gyrus	21	−78	45	2.07	0.001	126
	*Sulcal depth (left hemisphere)*						
CDs < Controls	Superior frontal gyrus	−47	10	17	3.15	0.004	220
	Caudal middle frontal gyrus	−23	−11	68	2.80	0.004	118
	*Sulcal depth (right hemisphere)*						
CDs > Controls	Lateral occipital gyrus	42	−84	−14	3.42	0.010	115
CDs < Controls	Rostral middle frontal gyrus	43	−60	9	2.39	0.024	173

N-parametric permutation testing with 5,000 permutations and threshold-free cluster enhancement (TFCE) with family-wise error corrected threshold of *p* < 0.05 was used.

#### Fractal dimensionality

The fractal dimensionality revealed bi-directional results. When compared to young HCs, the young CDs showed significant increases in the lateral occipital, lingual and insula gyri, as well as, the superior and inferior parietal lobules in the left hemisphere. Contrarily, significant decreases in fractal dimensionality were observed in the young CDs compared to the young HCs in the left superior temporal and superior frontal gyri, right superior and inferior parietal lobules, right precuneus, insula, parstriangularis, rostral middle frontal gyri, as well as the bilateral supramarginal and postcentral gyri (see Figure 1B and Table 2).

The fractal dimensionality also demonstrated bi-directional results between young CDs and old HCs. Compared to the old HCs, the young CDs exhibited significantly increased fractal dimensionality in the superior frontal, rostral and caudal middle frontal, lateral orbitofrontal, lingual, supramarginal and rostral anterior cingulate gyri, superior and paracentral lobules in the left hemisphere, and the right pericalcarine and medial orbitofrontal gyri in the right hemisphere. However, the young CDs also showed significantly decreased fractal dimensionality in the left inferior parietal lobule and the right superior frontal and parstriangularis gyri compared to the old HCs (see Figure 2B and Table 3).

#### Gyrification index

Compared to the young HCs, the young CDs illustrated significant increased gyrification in the superior frontal, rostral middle frontal, medial and lateral orbitofrontal, caudal and rostral anterior cingulate, isthmus cingulate, precentral, precuneus gyri, superior parietal, and paracentral lobules in the left hemisphere, as well as the supramarginal gyrus in the right hemisphere (see Figure 1C and Table 2).

Compared to the old HCs, the young CDs exhibited significantly increased gyrification in the posterior and caudal anterior cingulate gyri, and paracentral lobule in the left hemisphere, in the superior temporal, insula, rostral middle frontal and lateral orbitofrontal gyri in the right hemisphere, as well as significantly decreased gyrification in the right fusiform and lateral occipital gyri (see Figure 2C and Table 3).

#### Sulcal depth

Compared to the young HCs, the young CDs showed significantly increased sulcal depth only in the right inferior parietal lobule. They also revealed significantly decreased sulcal depth in the caudal middle frontal, cuneus, entorhinal gyri in the left hemisphere, the precentral, postcentral, supramarginal and middle temporal gyri, superior and inferior parietal lobules, and paracentral lobule in the right hemisphere, as well as the bilateral superior frontal and posterior cingulate gyri in the bilateral hemispheres (see Figure 1D and Table 2).

Compared to the old HCs, the young CDs presented with significantly increased sulcal depth only in the right lateral occipital gyrus, and exhibited significantly decreased sulcal depth in the left superior frontal and caudal middle frontal gyri, as well as the right rostral middle frontal gyrus (see Figure 2D and Table 3).

#### Correlation analysis

The correlation analysis was conducted to examine the relationship between the cortical morphology and VF raw score in each group. Table 4 shows that the CDs showed significant correlations between the VF score and the left supramarginal gyrus in fractal dimensionality, and the left caudal anterior cingulate, the left posterior cingulate, and precentral gyri in gyrification. The young HCs revealed significant correlations between the VF score and the left superior frontal gyrus in fractal dimensionality and the left superior frontal gyrus in gyrification.

**Table 4:** Correlation between verbal fluency raw score and cortical surface morphology.

Participants	Measures	Regions	*r*_(12)_	*p*
Young CDs	Fractal dimensionality	Left supramarginal gyrus	0.784	0.003
	Gyrification	Left caudal anterior cingulate gyrus	0.822	0.001
		Left precentral gyrus	0.716	0.009
		Left posterior cingulate gyrus	0.822	0.001
Young HCs	Fractal dimensionality	Left superior frontal gyrus	0.586	0.045
	Gyrification	Left superior frontal gyrus	0.586	0.045
Old HCs	Fractal dimensionality	Left lingual gyrus	0.655	0.021
		Left supramarginal gyrus	0.601	0.039
	Sulcal depth	Right lateral occipital gyrus	−0.619	0.032

Table 4 also illustrates the old HCs showed significant correlations between the VF score and the left lingual and supramarginal gyri in fractal dimensionality and the right lateral occipital gyrus in sulcal depth.

**Figure 3:**
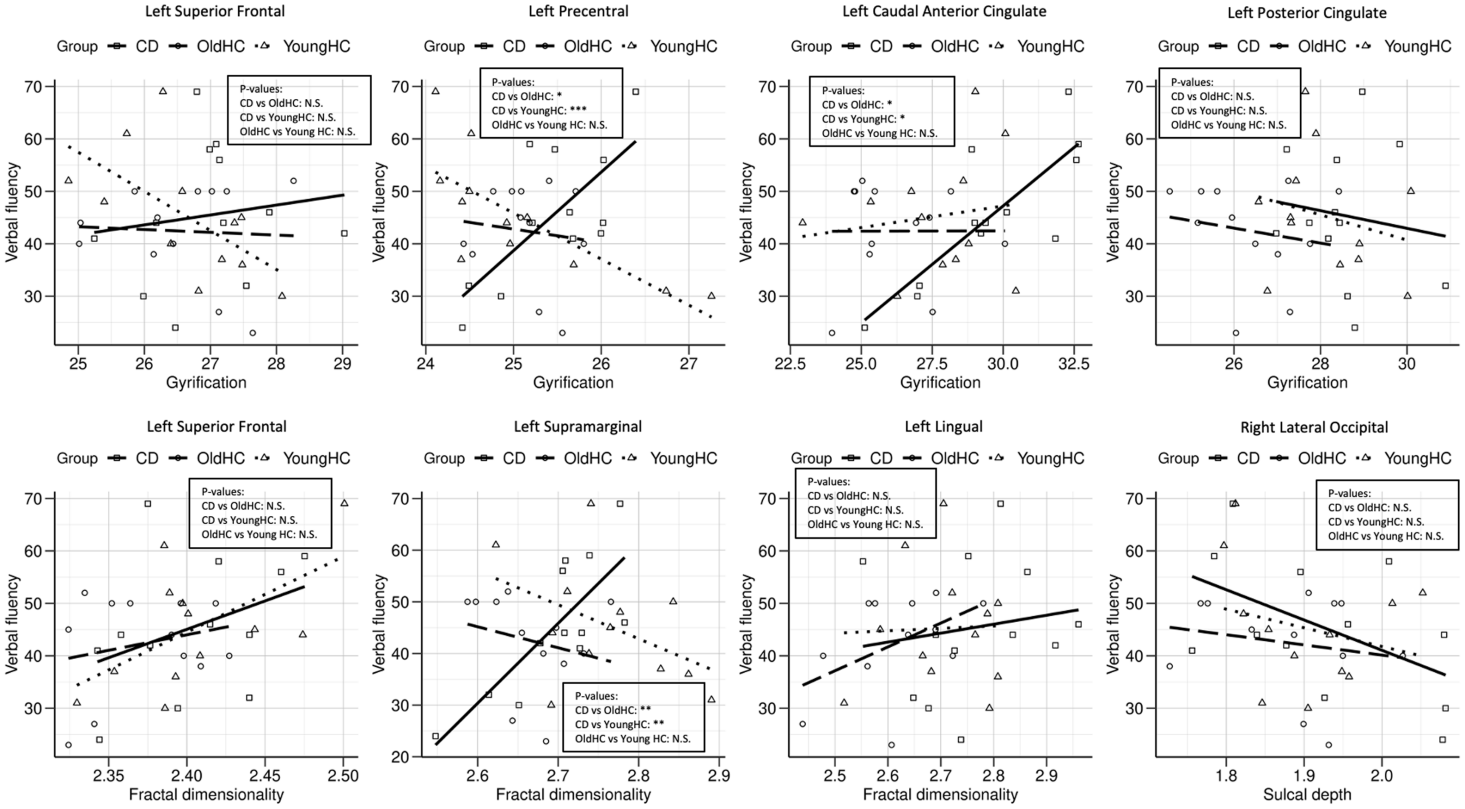
Group comparisons for all regions and cortical brain metrics with significant correlations with verbal fluency. Key: CD = Crohn’s disease patients, OldHC = old healthy controls, YoungHC = young healthy controls. *p*-values: N.S. = no significance, * = *p* < 0.05–0.01, ** = *p* < 0.01–0.001, *** = *p* < 0.001.

Figure 3 illustrates the correlation analysis across groups we conducted using Fisher’s r to z transformation to determine if the 3 groups had statistically significantly different relationships for a given cortical measurement, brain region, and VF score. This analysis was completed for the 8 combinations of cortical measurements and brain regions with significant correlations with VF seen in Table 4 to determine if there were dose–response effects between groups. Figure 3 demonstrated CDs had significantly different slopes for gyrification of the left precentral and caudal anterior gyri, as well as, fractal dimensionality of the left supramarginal gyrus compared to both young and old HCs. The remaining analyses were non-significant.

## Discussion

The current study reported notable differences in brain morphometry between the young CDs and both young and old HCs. There were numerous findings where CDs had decreases in cortical surface measures in some regions, but also increased measures in other regions compared to both young and old HCs, suggesting that CD does not just affect the brain in one particular direction and remodeling may be occurring.

On the whole, Tables 2, 3 demonstrated that CDs had more brain regions with differences in brain morphometry measures when compared to the young HCs (54 regions with differences) as compared to the old HCs (30 regions with differences), suggesting that CD may alter brain structure making it appear more similar to old HCs. For reference, S1a – S1d and S2a – S2d are Supplementary Tables of the brain regions with non-significant differences for Tables 2, 3, respectively. Additionally, Tables 2, 3 demonstrated CDs had atypical brain morphometries compared to both young and old HCs in key regions of well-described cognitive networks, such as the default mode network (DFM) and language function pathways. The DFM, a network negatively associated with attention and associated with states of day-dreaming and mindwandering, changes as a function of age and is thought to be partially responsible for cognitive decline and memory dysfunction seen in healthy aging populations (Tsvetanov et al., 2016; Chaovalitwongse et al., 2017; Staffaroni et al., 2018). Interestingly, our study demonstrated CDs have more regions associated with the DFM (such as the posterior cingulate gyrus and inferior parietal lobule) that are significantly different from young HCs as opposed to old HCs (17 vs. 8, respectively). While our study did not set out to assess DFM function or connectivity, based on these findings it appears that CDs have structures involved in the DFM that more closely resemble older HCs. While there is evidence that suggests CD has an impact on the DFM (Thomann et al., 2017; Kornelsen et al., 2020; Skrobisz et al., 2020), further research is needed to assess the effect of CD on DFM function and connectivity and if it might resemble a form of accelerated aging. Neural pathways involved in language function (such as the supramarginal and pars triangularis gyri and the inferior parietal lobe) also appear to have brain regions in the CDs that more closely resemble old HCs compared to young HCs. In Tables 2, 3, CDs have more regions associated with language function that are significantly different from young HCs as opposed to old HCs (11 vs. 5, respectively). However, our study did not find any differences in VF performance to suggest these atypical morphometries have an impact on performance. Further discussion of VF and brain morphometry is discussed later.

These findings of atypical brain morphometries of CDs appearing more similar to old HCs is inline with our previous study that found fMRI task activation patterns during a verbal fluency task were more similar among young CDs and healthy aging older HCs than the young HCs (Nair et al., 2019). Furthermore, in the present study CDs had increased FD in the left inferior parietal lobule and decreased FD in the left supramarginal gyrus compared to young HC, whereas, the association between CDs and old HCs were reversed in these brain regions. This possibly suggests that CDs are moving toward brain morphometry that resembles older HCs. However, there are a number of other significant differences in brain region morphometries between CDs and both young and old HCs that were significantly different in the same direction (i.e., CDs < both old and young HCs or CDs > both old and young in a given brain region), making associations based on individual brain regions difficult to interpret. Perhaps a study with a larger sample size can clarify these associations to assist with interpretation. Nevertheless, it is apparent that CDs exhibit different brain morphometry compared to HCs as demonstrated by previous studies (Zikou et al., 2014; Bao et al., 2015; Nair et al., 2016; Thomann et al., 2016; Bao et al., 2017a; Yeung, 2021; Thapaliya et al., 2022).

Current literature suggests that both the innate and adaptive immune system in CD are involved in altering intestinal mucosal permeability, making bacterial translocation and systemic inflammation possible, with interactions between host inflammation and microbiota implicated in disease progression (Petagna et al., 2020). Similarly, gut barrier dysfunction has been implicated in bacterial translocation and organ failure in a variety of diseases such as Grave’s disease (Zheng et al., 2021), acute pancreatitis (Li et al., 2020), hepatic disorders (Chopyk and Grakoui, 2020), stress and mood disorders (Doney et al., 2022), and Alzheimer’s disease (Megur et al., 2020; Liu et al., 2021). In addition, gut barrier dyfunction may be a primary driver of systemic inflammation and organ failure observed in the elderly population (Deitch, 1990). Not only this, but there is evidence that elderly patients may not have an increased strength of the inflammatory response, but a more protracted response that is responsible for poorer outcomes during similar pathologic insults in an older population as compared to their younger counterparts (Fagiolo et al., 1993; Kudoh et al., 2001; Pinheiro da Silva et al., 2013; Ren et al., 2014). This protracted inflammatory response seen in older patients is not too dissimilar to the chronicity seen in CD, and one could argue the chronicity may even be more pronounced in the CD population given its lifelong recurring and remitting course. Furthermore, recent studies suggest certain inflammatory markers, such as IL18R1, demonstrate a causal relationship with both IBD and pathologies of the aging brain, such as Alzheimer’s disease (Hillary et al., 2020), further demonstrating a link between IBD and aging brain function.

To investigate the effects CD has on brain function our study had CDs and HCs complete a VF task to explore differences in cognitive function between groups. Although there were no group differences, there was an association between better performance on the VF task and the FD in the left supramarginal gyrus in both the CDs and old HCs that was not present in the young HCs group. There were no overlapping associations between brain morphometry and VF task performance between CDs and young HCs. With performance remaining the same across groups, this may suggest a shift in function compensation by the CDs that more similarly resembles that of the old HCs.

However, Figure 3 (VF vs. FD supramarginal) demonstrates CDs have significantly different correlations for supramarginal fractal dimensionality and VF performance compared to both young and old HCs where increasing fractal dimensionality is associated with better VF for CDs, contradicting this assertion. Additionally, gyrification of both the left caudal anterior cingulate and left precentral gyrus have significantly different correlations with VF performance for CDs compared to both young and old HCs where increasing gyrification is associated with better VF. With the remaining brain regions and cortical measures for CDs, Young HCs, and old HCs that were associated with VF performance not having statistically significantly different slopes among groups, it appears as though CDs may have a different adaptation pattern for performing the VF task as compared to both old and young HCs. In fact, 6 out of the 8 comparisons in Figure 3 demonstrated a positive relationship with CDs compared to 3 out of the 8 for both healthy control groups. This seems to suggest CDs recruit more brain regions in order to perform the same VF task as compared to both young and old HCs. Its possbile these differences are a result of the varying medications CDs require to combat the disease process or a result of the disease process itself, but a causal relationship is not assessable within the constraints of the present study. Lastly, with the limited sample size of our study, it is possible that the lack of positive associations seen with VF performance in the young and old HCs is an artifact of the study and perhaps, further research with a larger study population will help elucidate more measures and regions of significance that can assist with interpretation of these findings.

Interestingly, the HAROLD (hemispheric asymmetry reduction in older adults) model of hemispheric aging was not demonstrated in the CDs VF performance, but was identified in the old HCs; with the old HCs having VF performance correlate with sulcal depth of the right lateral occipital gyrus, whereas a left lateralization of language performance is the predominant finding in both CDs and young HCs (Cabeza, 2002). Perhaps these findings are a result of CDs not having progressed as far on the bi-hemispheric pattern of aging timeline or have not had enough time to develop new brain response patterns involving this brain region. Lastly, given that studies have shown that education, employment, and income are not significantly different between patients with IBD and healthy individuals (El-Matary et al., 2017), it is possible that CD patients might adopt adaptive cognitive strategies to maintain function despite structural and functional brain changes resulting from this lifelong disease. Although the investigation of brain changes in CD patients has become an increasing focus of several recent studies to explore brain-gut interactions (Thomann et al., 2017; Peppas et al., 2021), our study demonstrates that atypical brain morphometry of CDs is more similar to old HCs and this atypical brain morphometry is associated with function on a cognitive task. These results suggest that even younger CDs may be showing some evidence of structural brain changes that demonstrate increased resemblance to older HC brains rather than their similarly aged healthy counterparts. However, the current study demonstrated that these structural brain changes did not result in similar brain response patterns on a cognitive task as compared to young or old HCs. Future longitudinal studies will be needed in order to better understand the effect CD has on brain structure and function over time and whether or not it resembles a form of accelerated aging.

The modest sample size is a limitation and the results can be substantiated with adequately powered future studies. All of our CDs were on treatments with at least one standard IBD maintenance medication; however, the number and combination of medications they were taking, as well as the classes of those medications, varied among participants. Differences in medication regimens might have influenced brain morphometry or task performance. The duration of the disease and the age at CD diagnosis also varied among patients, which could have contributed to the changes observed in their brain morphometry.

## Data availability statement

The raw data supporting the conclusions of this article will be made available by the authors, without undue reservation.

## Ethics statement

The studies involving humans were approved by University of Wisconsin Institutional Review Board. The studies were conducted in accordance with the local legislation and institutional requirements. The participants provided their written informed consent to participate in this study.

## Author contributions

SS was responsible for funding acquisition. VP, PB-P, SS, and VN conceived and designed the experiments. VN, PB-P, and SS helped with data acquisition. JH preprocessed the data and wrote the manuscript methods and results section. BY, SS, and JH analyzed the data. BY wrote the intro and discussion of the manuscript. VN, NA, DC, PB-P, SS, and VP provided guidance for data analysis, and manuscript writing and editing. All authors contributed to the article and approved the submitted version.

## Footnotes

^1^ http://www.neuro.uni-jena.de/cat/

^2^ https://www.fil.ion.ucl.ac.uk/spm/software/spm12/
