# Supplementary material for: Structural brain morphometry differences and similarities between young patients with Crohn’s disease in remission and healthy young and old controls
Source: Front Neurosci. 2024 Jan 31;18:1210939. doi: 10.3389/fnins.2024.1210939 (PMC10864509; doi:10.3389/fnins.2024.1210939)
Supplement: Supplementary file 1 [file Table_1.DOCX]

**S1 (a-d):** All non-significant brain region and cortical surface morphology for the comparison of young CD vs young HC.

1. **Thickness**

| **Regions** | ***t*_(22)_** | ***p*** |
| --- | --- | --- |
| left caudal anterior cingulate | -0.215 | 0.832 |
| right caudal anterior cingulate | -0.633 | 0.534 |
| left caudal middle frontal | -0.819 | 0.421 |
| right caudal middle frontal | -0.093 | 0.927 |
| left cuneus | 0.701 | 0.491 |
| right cuneus | 0.262 | 0.796 |
| left entorhinal | 0.206 | 0.839 |
| right entorhinal | 0.163 | 0.872 |
| left fusiform | -0.282 | 0.781 |
| left inferior parietal | -0.367 | 0.717 |
| right inferior parietal | -0.514 | 0.612 |
| left inferior temporal | 0.135 | 0.894 |
| right inferior temporal | 0.411 | 0.685 |
| left isthmus cingulate | 0.723 | 0.477 |
| right isthmus cingulate | 1.603 | 0.123 |
| left lateral occipital | 0.269 | 0.791 |
| left lateral orbitofrontal | 0.484 | 0.633 |
| right lateral orbitofrontal | -0.389 | 0.701 |
| left lingual | -0.056 | 0.956 |
| left medial orbitofrontal | 0.202 | 0.842 |
| right medial orbitofrontal | 1.141 | 0.266 |
| left middle temporal | 0.691 | 0.497 |
| right middle temporal | 1.392 | 0.178 |
| left parahippocampal | 0.061 | 0.952 |
| right parahippocampal | 0.242 | 0.811 |
| left paracentral | -1.51 | 0.192 |
| right paracentral | -0.954 | 0.351 |
| left pars opercularis | -1.833 | 0.08 |
| right pars opercularis | 0.062 | 0.951 |
| left pars orbitalis | 1.179 | 0.251 |
| right pars orbitalis | 0.334 | 0.742 |
| left pars triangularis | -0.509 | 0.616 |
| right pars triangularis | 0.419 | 0.679 |
| left pericalcarine | 0.902 | 0.377 |
| right pericalcarine | 0.055 | 0.957 |
| left postcentral | 0.478 | 0.638 |
| right postcentral | -0.088 | 0.93 |
| left posterior cingulate | -0.947 | 0.354 |
| right posterior cingulate | 0.305 | 0.763 |
| left precentral | -0.194 | 0.848 |
| right precentral | 0.135 | 0.894 |
| left precuneus | 0.372 | 0.714 |
| right precuneus | 0.434 | 0.668 |
| left rostral anterior cingulate | 2.021 | 0.056 |
| right rostral anterior cingulate | 0.581 | 0.567 |
| left rostral middle frontal | -0.913 | 0.371 |
| right rostral middle frontal | -0.001 | 0.999 |
| left superior frontal | 0.885 | 0.385 |
| right superior frontal | 0.335 | 0.741 |
| left superior parietal | -0.068 | 0.946 |
| right superior parietal | -0.453 | 0.655 |
| left superior temporal | 0.933 | 0.361 |
| right superior temporal | 1.621 | 0.119 |
| left supramarginal | -0.033 | 0.974 |
| right supramarginal | -0.202 | 0.841 |
| left frontal pole | 1.413 | 0.172 |
| right frontal pole | 0.421 | 0.678 |
| left temporal pole | -0.515 | 0.611 |
| right temporal pole | -0.595 | 0.558 |
| left transverse temporal | -1.162 | 0.252 |
| right transverse temporal | 0.959 | 0.348 |
| left insula | -1.085 | 0.289 |
| right insula | 0.802 | 0.431 |

1. **Fractal dimensionality**

| **Regions** | ***t*_(22)_** | ***p*** |
| --- | --- | --- |
| left caudal anterior cingulate | -1.686 | 0.106 |
| right caudal anterior cingulate | 0.335 | 0.741 |
| left caudal middle frontal | 1.313 | 0.203 |
| right caudal middle frontal | -0.521 | 0.608 |
| left cuneus | 0.092 | 0.928 |
| right cuneus | -0.492 | 0.627 |
| left entorhinal | 0.656 | 0.518 |
| right entorhinal | -0.537 | 0.597 |
| left fusiform | 1.327 | 0.198 |
| right fusiform | 0.378 | 0.709 |
| left inferior temporal | -0.531 | 0.6 |
| right inferior temporal | -0.07 | 0.945 |
| left isthmus cingulate | -0.03 | 0.976 |
| right isthmus cingulate | 0.696 | 0.494 |
| right lateral occipital | -0.222 | 0.826 |
| left lateral orbitofrontal | 0.145 | 0.886 |
| right lateral orbitofrontal | 1.024 | 0.317 |
| right lingual | 1.085 | 0.29 |
| left medial orbitofrontal | -0.202 | 0.841 |
| right medial orbitofrontal | 1.514 | 0.144 |
| left middle temporal | -0.036 | 0.972 |
| right middle temporal | 0.94 | 0.358 |
| left parahippocampal | 0.024 | 0.981 |
| right parahippocampal | -0.213 | 0.833 |
| left paracentral | -0.097 | 0.923 |
| right paracentral | -0.038 | 0.97 |
| left pars opercularis | -0.746 | 0.463 |
| right pars opercularis | -0.255 | 0.801 |
| left pars orbitalis | 0.389 | 0.701 |
| right pars orbitalis | 1.071 | 0.296 |
| left pars triangularis | -0.781 | 0.443 |
| left pericalcarine | -0.349 | 0.73 |
| right pericalcarine | 1.216 | 0.237 |
| left posterior cingulate | 0.082 | 0.936 |
| right posterior cingulate | 0.512 | 0.614 |
| left precentral | -1.249 | 0.225 |
| right precentral | -0.909 | 0.373 |
| left precuneus | -1.12 | 0.275 |
| left rostral anterior cingulate | 1.182 | 0.25 |
| right rostral anterior cingulate | -0.897 | 0.379 |
| left rostral middle frontal | 0.393 | 0.698 |
| right superior frontal | -0.34 | 0.737 |
| right superior temporal | -1.67 | 0.109 |
| left frontal pole | -0.693 | 0.495 |
| right frontal pole | 0.682 | 0.503 |
| left temporal pole | -1.969 | 0.062 |
| right temporal pole | 0.413 | 0.684 |
| left transverse temporal | -0.891 | 0.383 |
| right transverse temporal | -0.903 | 0.376 |

1. **Gyrification**

| **Regions** | ***t*_(22)_** | ***p*** |
| --- | --- | --- |
| right caudal anterior cingulate | 0.546 | 0.591 |
| left caudal middle frontal | -0.29 | 0.775 |
| right caudal middle frontal | 1.087 | 0.289 |
| left cuneus | 0.558 | 0.582 |
| right cuneus | -0.356 | 0.725 |
| left entorhinal | -0.777 | 0.446 |
| right entorhinal | -0.043 | 0.966 |
| left fusiform | 0.534 | 0.599 |
| right fusiform | -1.621 | 0.119 |
| left inferior parietal | 1.267 | 0.219 |
| right inferior parietal | 0.596 | 0.557 |
| left inferior temporal | 0.002 | 0.998 |
| right inferior temporal | -0.616 | 0.544 |
| right isthmus cingulate | -0.228 | 0.822 |
| left lateral occipital | -0.9 | 0.378 |
| right lateral occipital | -0.416 | 0.682 |
| right lateral orbitofrontal | 0.601 | 0.554 |
| left lingual | -0.222 | 0.826 |
| right lingual | 1.259 | 0.221 |
| right medial orbitofrontal | -0.773 | 0.448 |
| left middle temporal | -0.293 | 0.773 |
| right middle temporal | -1.133 | 0.269 |
| left parahippocampal | 0.596 | 0.557 |
| right parahippocampal | -0.868 | 0.395 |
| right paracentral | 0.606 | 0.551 |
| left pars opercularis | -0.028 | 0.978 |
| right pars opercularis | 0.807 | 0.429 |
| left pars orbitalis | 0.784 | 0.441 |
| right pars orbitalis | 1.074 | 0.294 |
| left pars triangularis | 0.095 | 0.925 |
| right pars triangularis | 1.241 | 0.228 |
| left pericalcarine | -0.781 | 0.443 |
| right pericalcarine | -0.96 | 0.347 |
| left posterior cingulate | 0.929 | 0.363 |
| right posterior cingulate | -0.285 | 0.779 |
| right precentral | 0.461 | 0.65 |
| right precuneus | 0.426 | 0.674 |
| right rostral anterior cingulate | 1.142 | 0.266 |
| right rostral middle frontal | 0.008 | 0.993 |
| right superior frontal | -0.628 | 0.537 |
| right superior parietal | 0.699 | 0.492 |
| left superior temporal | -0.984 | 0.336 |
| right superior temporal | 0.428 | 0.673 |
| left supramarginal | -0.674 | 0.507 |
| left frontal pole | -0.6 | 0.555 |
| right frontal pole | -0.805 | 0.429 |
| left temporal pole | -0.258 | 0.799 |
| right temporal pole | -1.631 | 0.117 |
| left transverse temporal | 1.000 | 0.328 |
| right transverse temporal | 1.951 | 0.064 |
| left insula | 0.079 | 0.938 |
| right insula | -1.433 | 0.166 |

1. **Sulcal depth**

| **Regions** | ***t*_(22)_** | ***p*** |
| --- | --- | --- |
| left caudal anterior cingulate | 0.145 | 0.886 |
| right caudal anterior cingulate | -0.424 | 0.676 |
| right caudal middle frontal | -0.339 | 0.738 |
| right cuneus | -2.266 | 0.034 |
| right entorhinal | -1.07 | 0.986 |
| left fusiform | -0.748 | 0.462 |
| right fusiform | 0.185 | 0.855 |
| left inferior parietal | 0.169 | 0.868 |
| left inferior temporal | 0.88 | 0.388 |
| right inferior temporal | -0.375 | 0.712 |
| left isthmus cingulate | -0.839 | 0.411 |
| right isthmus cingulate | -0.083 | 0.935 |
| left lateral occipital | -0.308 | 0.761 |
| right lateral occipital | 0.532 | 0.6 |
| left lateral orbitofrontal | -1.02 | 0.319 |
| right lateral orbitofrontal | -0.37 | 0.715 |
| left lingual | -1.455 | 0.16 |
| right lingual | -0.093 | 0.927 |
| left medial orbitofrontal | -1.141 | 0.364 |
| right medial orbitofrontal | 1.955 | 0.063 |
| left middle temporal | -0.367 | 0.717 |
| left parahippocampal | -1.403 | 0.174 |
| right parahippocampal | -0.188 | 0.853 |
| left paracentral | -1.29 | 0.21 |
| left pars opercularis | 0.981 | 0.337 |
| right pars opercularis | -0.223 | 0.826 |
| left pars orbitalis | 0.424 | 0.675 |
| right pars orbitalis | 0.22 | 0.828 |
| left pars triangularis | 0.455 | 0.653 |
| right pars triangularis | 1.123 | 0.274 |
| left pericalcarine | 0.244 | 0.809 |
| right pericalcarine | -0.681 | 0.503 |
| left postcentral | -1.126 | 0.272 |
| left precentral | 0.014 | 0.989 |
| left precuneus | -1.866 | 0.075 |
| right precuneus | -0.991 | 0.332 |
| left rostral anterior cingulate | -0.437 | 0.666 |
| right rostral anterior cingulate | 0.88 | 0.389 |
| left rostral middle frontal | -0.025 | 0.98 |
| right rostral middle frontal | 0.041 | 0.967 |
| left superior parietal | 0.859 | 0.4 |
| left superior temporal | -0.491 | 0.628 |
| right superior temporal | -1.054 | 0.303 |
| left supramarginal | -0.551 | 0.587 |
| left frontal pole | -0.594 | 0.558 |
| right frontal pole | -0.241 | 0.812 |
| left temporal pole | -0.324 | 0.749 |
| right temporal pole | -1.107 | 0.28 |
| left transverse temporal | 0.089 | 0.93 |
| right transverse temporal | -1.018 | 0.32 |
| left insula | -0.628 | 0.537 |
| right insula | -0.952 | 0.352 |
