# Supplementary material for: Structural brain morphometry differences and similarities between young patients with Crohn’s disease in remission and healthy young and old controls
Source: Front Neurosci. 2024 Jan 31;18:1210939. doi: 10.3389/fnins.2024.1210939 (PMC10864509; doi:10.3389/fnins.2024.1210939)
Supplement: Supplementary file 2 [file Table_2.DOCX]

**S2 (a-d):** All non-significant brain region and cortical surface morphology for the comparison of young CD vs young HC.

1. **Thickness**

| **Regions** | ***t*_(22)_** | ***p*** |
| --- | --- | --- |
| left caudal anterior cingulate | 1.123 | 0.212 |
| right caudal anterior cingulate | 1.75 | 0.094 |
| left caudal middle frontal | 1.93 | 0.067 |
| right caudal middle frontal | -0.401 | 0.692 |
| left cuneus | -0.786 | 0.44 |
| right cuneus | 0.05 | 0.961 |
| left entorhinal | -0.605 | 0.551 |
| right entorhinal | 1.33 | 0.197 |
| left fusiform | 0.209 | 0.836 |
| right fusiform | 0.69 | 0.497 |
| left inferior parietal | -0.296 | 0.77 |
| right inferior parietal | 0.791 | 0.437 |
| left inferior temporal | -0.098 | 0.923 |
| right inferior temporal | 0.263 | 0.795 |
| left isthmus cingulate | -0.092 | 0.927 |
| right isthmus cingulate | 1.011 | 0.323 |
| left lateral occipital | 1.459 | 0.159 |
| right lateral occipital | -0.132 | 0.896 |
| left lateral orbitofrontal | 0.172 | 0.865 |
| right lateral orbitofrontal | 1.585 | 0.127 |
| left lingual | 0.383 | 0.705 |
| right lingual | 0.137 | 0.892 |
| left medial orbitofrontal | -0.308 | 0.761 |
| right medial orbitofrontal | 0.585 | 0.564 |
| left middle temporal | 0.078 | 0.938 |
| right middle temporal | 1.226 | 0.233 |
| left parahippocampal | 1.364 | 0.187 |
| right parahippocampal | 0.413 | 0.683 |
| left paracentral | 0.545 | 0.592 |
| right paracentral | 1.338 | 0.194 |
| left pars opercularis | -0.12 | 0.906 |
| right pars opercularis | 1.191 | 0.246 |
| left pars orbitalis | -1.169 | 0.255 |
| right pars orbitalis | 1.001 | 0.328 |
| left pars triangularis | 0.674 | 0.507 |
| right pars triangularis | -0.68 | 0.503 |
| left pericalcarine | -0.318 | 0.754 |
| right pericalcarine | -0.78 | 0.443 |
| left postcentral | -0.415 | 0.682 |
| left posterior cingulate | -0.891 | 0.382 |
| right posterior cingulate | -1.151 | 0.262 |
| left precentral | 1.269 | 0.172 |
| right precentral | -0.244 | 0.809 |
| left precuneus | -1.691 | 0.105 |
| right precuneus | -0.492 | 0.628 |
| left rostral anterior cingulate | 0.508 | 0.616 |
| right rostral anterior cingulate | -0.218 | 0.83 |
| left rostral middle frontal | -0.728 | 0.474 |
| right rostral middle frontal | 0.863 | 0.398 |
| left superior frontal | 0.357 | 0.725 |
| right superior frontal | 0.695 | 0.495 |
| left superior parietal | 0.349 | 0.731 |
| right superior parietal | 0.423 | 0.676 |
| left superior temporal | 0.251 | 0.804 |
| right superior temporal | -0.585 | 0.565 |
| left supramarginal | 0.355 | 0.726 |
| right supramarginal | 0.545 | 0.591 |
| left frontal pole | 0.658 | 0.518 |
| right frontal pole | 0.951 | 0.352 |
| left temporal pole | 0.341 | 0.736 |
| right temporal pole | 0.524 | 0.605 |
| left transverse temporal | -0.006 | 0.995 |
| right transverse temporal | 0.409 | 0.687 |
| left insula | 1.327 | 0.198 |
| right insula | 0.347 | 0.732 |

1. **Fractal dimensionality**

| **Regions** | ***t*_(22)_** | ***p*** |
| --- | --- | --- |
| left caudal anterior cingulate | -1.003 | 0.327 |
| right caudal anterior cingulate | 1.749 | 0.094 |
| right caudal middle frontal | 0.268 | 0.791 |
| left cuneus | 1.357 | 0.189 |
| right cuneus | -0.165 | 0.87 |
| left entorhinal | 0.365 | 0.719 |
| right entorhinal | -1.118 | 0.276 |
| left fusiform | 0.146 | 0.885 |
| right fusiform | 1.577 | 0.129 |
| right inferior parietal | 1.685 | 0.106 |
| left inferior temporal | 0.257 | 0.799 |
| right inferior temporal | -0.125 | 0.902 |
| left isthmus cingulate | -0.101 | 0.921 |
| right isthmus cingulate | 1.049 | 0.306 |
| left lateral occipital | -1.586 | 0.127 |
| right lateral occipital | -0.389 | 0.701 |
| right lateral orbitofrontal | 1.589 | 0.126 |
| right lingual | 0.442 | 0.662 |
| left medial orbitofrontal | 1.041 | 0.309 |
| left middle temporal | -0.086 | 0.932 |
| right middle temporal | 1.163 | 0.257 |
| left parahippocampal | 0.011 | 0.991 |
| right parahippocampal | -0.633 | 0.534 |
| right paracentral | -0.351 | 0.729 |
| left pars opercularis | -0.701 | 0.491 |
| right pars opercularis | -0.164 | 0.871 |
| left pars orbitalis | -0.107 | 0.916 |
| right pars orbitalis | -1.268 | 0.218 |
| left pars triangularis | -0.962 | 0.346 |
| left pericalcarine | -0.606 | 0.551 |
| left postcentral | 0.565 | 0.578 |
| right postcentral | 0.508 | 0.616 |
| left posterior cingulate | -1.175 | 0.239 |
| right posterior cingulate | 0.136 | 0.893 |
| left precentral | -0.214 | 0.833 |
| right precentral | -0.285 | 0.778 |
| left precuneus | 0.672 | 0.509 |
| right precuneus | 1.398 | 0.176 |
| right rostral anterior cingulate | 1.157 | 0.26 |
| right rostral middle frontal | 0.307 | 0.762 |
| right superior parietal | 0.135 | 0.894 |
| left superior temporal | 0.696 | 0.494 |
| right superior temporal | 0.395 | 0.697 |
| right supramarginal | -1.134 | 0.269 |
| left frontal pole | -0.404 | 0.69 |
| right frontal pole | 1.012 | 0.323 |
| left temporal pole | -0.395 | 0.697 |
| right temporal pole | 1.218 | 0.236 |
| left transverse temporal | -0.607 | 0.55 |
| right transverse temporal | -1.139 | 0.267 |
| left insula | 1.13 | 0.271 |
| right insula | -0.035 | 0.972 |

1. **Gyrification**

| **Regions** | ***t*_(22)_** | ***p*** |
| --- | --- | --- |
| right caudal anterior cingulate | 1.775 | 0.09 |
| left caudal middle frontal | -0.224 | 0.825 |
| right caudal middle frontal | 1.495 | 0.149 |
| left cuneus | 0.236 | 0.816 |
| right cuneus | -0.12 | 0.905 |
| left entorhinal | -0.278 | 0.784 |
| right entorhinal | 1.651 | 0.271 |
| left fusiform | 0.562 | 0.58 |
| left inferior parietal | -1.498 | 0.148 |
| right inferior parietal | -1.939 | 0.065 |
| left inferior temporal | -0.902 | 0.377 |
| right inferior temporal | -1.23 | 0.232 |
| left isthmus cingulate | 1.034 | 0.312 |
| right isthmus cingulate | 0.52 | 0.609 |
| left lateral occipital | -0.243 | 0.81 |
| left lateral orbitofrontal | -0.051 | 0.96 |
| left lingual | -0.852 | 0.403 |
| right lingual | 0.512 | 0.614 |
| left medial orbitofrontal | 0.69 | 0.498 |
| right medial orbitofrontal | -1.193 | 0.245 |
| left middle temporal | -1.887 | 0.072 |
| right middle temporal | -0.834 | 0.413 |
| left parahippocampal | -0.534 | 0.599 |
| right parahippocampal | -0.402 | 0.692 |
| right paracentral | 1.19 | 0.247 |
| left pars opercularis | 1.194 | 0.245 |
| right pars opercularis | 1.927 | 0.187 |
| left pars orbitalis | -0.244 | 0.809 |
| right pars orbitalis | 0.888 | 0.384 |
| left pars triangularis | -0.148 | 0.884 |
| right pars triangularis | 1.437 | 0.165 |
| left pericalcarine | -0.284 | 0.779 |
| right pericalcarine | -0.703 | 0.489 |
| left postcentral | -0.11 | 0.913 |
| right postcentral | 0.482 | 0.635 |
| right posterior cingulate | 0.781 | 0.443 |
| left precentral | 1.341 | 0.194 |
| right precentral | 1.653 | 0.113 |
| left precuneus | 0.622 | 0.54 |
| right precuneus | 0.224 | 0.825 |
| left rostral anterior cingulate | 1.267 | 0.218 |
| right rostral anterior cingulate | 0.852 | 0.404 |
| left rostral middle frontal | 0.117 | 0.908 |
| left superior frontal | 0.994 | 0.331 |
| right superior frontal | 1.156 | 0.26 |
| left superior parietal | -0.696 | 0.494 |
| right superior parietal | 1.958 | 0.063 |
| left superior temporal | -0.528 | 0.603 |
| left supramarginal | -1.193 | 0.245 |
| right supramarginal | 0.755 | 0.458 |
| left frontal pole | -0.755 | 0.458 |
| right frontal pole | 0.393 | 0.698 |
| left temporal pole | 0.3 | 0.767 |
| right temporal pole | -0.495 | 0.626 |
| left transverse temporal | 1.2 | 0.243 |
| right transverse temporal | 1.224 | 0.234 |
| left insula | 0.513 | 0.613 |

1. **Sulcal depth**

| **Regions** | ***t*_(22)_** | ***p*** |
| --- | --- | --- |
| left caudal anterior cingulate | -0.117 | 0.908 |
| right caudal anterior cingulate | 0.104 | 0.918 |
| right caudal middle frontal | 0.239 | 0.813 |
| left cuneus | -0.025 | 0.981 |
| right cuneus | -0.502 | 0.621 |
| left entorhinal | -1.157 | 0.26 |
| right entorhinal | -0.528 | 0.603 |
| left fusiform | -0.09 | 0.929 |
| right fusiform | 0.299 | 0.768 |
| left inferior parietal | 0.338 | 0.738 |
| right inferior parietal | 1.908 | 0.07 |
| left inferior temporal | 0.186 | 0.854 |
| right inferior temporal | 0.467 | 0.645 |
| left isthmus cingulate | 1.259 | 0.221 |
| right isthmus cingulate | 0.499 | 0.623 |
| left lateral occipital | 0.515 | 0.612 |
| left lateral orbitofrontal | -0.104 | 0.918 |
| right lateral orbitofrontal | -1.1 | 0.283 |
| left lingual | 0.47 | 0.643 |
| right lingual | -0.081 | 0.936 |
| left medial orbitofrontal | -1.379 | 0.182 |
| right medial orbitofrontal | 0.898 | 0.379 |
| left middle temporal | 1.419 | 0.17 |
| right middle temporal | 0.605 | 0.551 |
| left parahippocampal | -0.642 | 0.528 |
| right parahippocampal | -0.552 | 0.587 |
| left paracentral | -0.587 | 0.563 |
| right paracentral | -1.042 | 0.309 |
| left pars opercularis | 1.339 | 0.194 |
| right pars opercularis | 0.939 | 0.358 |
| left pars orbitalis | 0.957 | 0.349 |
| right pars orbitalis | -1.254 | 0.223 |
| left pars triangularis | 1.118 | 0.276 |
| right pars triangularis | 0.915 | 0.37 |
| left pericalcarine | 0.322 | 0.75 |
| right pericalcarine | -0.419 | 0.679 |
| left postcentral | -1.041 | 0.309 |
| right postcentral | -0.709 | 0.486 |
| left posterior cingulate | 1.013 | 0.322 |
| right posterior cingulate | 0.173 | 0.864 |
| left precentral | 0.425 | 0.675 |
| right precentral | -0.177 | 0.861 |
| left precuneus | 0.605 | 0.552 |
| right precuneus | 0.628 | 0.536 |
| left rostral anterior cingulate | 0.861 | 0.399 |
| right rostral anterior cingulate | 0.426 | 0.674 |
| left rostral middle frontal | -1.585 | 0.127 |
| right superior frontal | -0.945 | 0.355 |
| left superior parietal | 0.303 | 0.765 |
| right superior parietal | 0.13 | 0.897 |
| left superior temporal | 0.557 | 0.583 |
| right superior temporal | 0.976 | 0.34 |
| left supramarginal | -0.555 | 0.584 |
| right supramarginal | 0.26 | 0.797 |
| left frontal pole | 0.001 | 0.999 |
| right frontal pole | 0.752 | 0.46 |
| left temporal pole | -0.933 | 0.361 |
| right temporal pole | -0.254 | 0.802 |
| left transverse temporal | 0.855 | 0.402 |
| right transverse temporal | -0.03 | 0.977 |
| left insula | 0.953 | 0.351 |
| right insula | 0.353 | 0.727 |
